# Exploring the Intersection of Nursing Leadership and Artificial Intelligence: Scoping Review

**DOI:** 10.2196/80085

**Published:** 2025-11-14

**Authors:** Jessica S Burford, Richard G Booth, Amanda McIntyre

**Affiliations:** 1 Arthur Labatt Family School of Nursing, Faculty of Health Sciences, Western University, Room 3306, FIMS & Nursing Building, 1151 Richmond Street, London, ON, N6A 5B9, Canada, 1 519-661-3395

**Keywords:** nursing leadership, artificial intelligence: AI technology, scoping review, digital health, work environment, leadership development

## Abstract

**Background:**

As artificial intelligence (AI) technology permeates health care settings, nurse leaders must position themselves to shape its development, implementation, and impact, guiding meaningful change that benefits nurses and care delivery. Nurse leaders possess the capacity to influence decisions, shape practice, and ensure the delivery of ethical, safe, and high-quality care. While AI technology is reshaping many aspects of health care delivery, there is limited knowledge on how nurse leaders perceive and experience this shift.

**Objective:**

This scoping review aimed to explore the intersection of nursing leadership and AI technology in health care by mapping current evidence, identifying key concepts, and highlighting knowledge gaps within the literature.

**Methods:**

This scoping review was guided by the Joanna Briggs Institute methodology and reported on using the PRISMA-ScR (Preferred Reporting Items for Systematic Reviews and Meta-Analyses Extension for Scoping Reviews) checklist. A systematic search of 4 electronic databases (CINAHL [EBSCO Information Services], Ovid MEDLINE [Wolters Kluwer], PsycINFO [American Psychological Association], and Scopus [Elsevier]) was conducted for English-language, peer-reviewed literature published between 2014 and 2025. Gray literature was also reviewed. Articles were included if they met the inclusion criteria by exploring the population of nurse leaders and the concept of AI technology within the context of health care settings and were published in English from May 2014 forward. A total of 26 articles were included in the analysis. Qualitative content analysis and numerical summary supported the inductive identification and synthesis of data categories.

**Results:**

Of the 26 articles included, 8 were empirical (qualitative, quantitative, or mixed methods), and 18 were conceptual or theoretical articles. Although 1 article was Canadian, there were no empirical studies conducted by Canadian researchers. The qualitative content analysis of the primary search findings revealed 6 overarching data categories: (1) leading digital transformation and technology integration, (2) AI technology and the nursing role: reshaping practice, (3) ethical considerations of AI technology for nurse leaders, (4) AI technology as a facilitator of innovative leadership, (5) education and training on AI technology in nursing practice, and (6) influence of AI technology on the work environment.

**Conclusions:**

This review confirms that nurse leaders play an essential role in shaping the future of health care in the context of AI technology. Although this review highlights a growing recognition of nursing leadership as a crucial driver of AI technology integration in health care, there is a lack of research to guide practice, policy, and leadership development through education, despite emerging interest and a recent increase in empirical work. The findings accentuate the need for increased investment in nurse-led research and leadership development to ensure that AI systems are designed, implemented, and evaluated in a manner that upholds ethical care, equity, and professional nursing values. As health care systems increasingly adopt AI technology, nurse leaders must be equipped with the knowledge, tools, and support required to lead transformative change and act as AI technology directors.

## Introduction

### Background

Artificial intelligence (AI) is a rapidly developing technology that is augmenting health care delivery in multiple ways, as reflected in an increase in research over the past decade [1]. For the purposes of this scoping review, AI technology is defined as technology that allows computers and machines to mimic human cognitive functions, such as learning, understanding, problem-solving, decision-making, creativity, and autonomous aspects [2]. Despite the evolving impact of AI technology on nursing practice, few investigations have explored its intersection with nursing leadership. Nurse leaders have tremendous influence on moving teams toward a common goal [3] and instigating practice change. Understanding the nuanced and developing area of an AI technology–informed nursing leadership practice is necessary as the healthcare environment continues to shift.

To date, discourse on AI technology has centered on its potential to augment care delivery and improve patient outcomes [4]. However, few studies have critically examined how AI technology might impact nursing leadership roles or the extent to which nurse leaders are actively participating in decision-making and design processes involving AI technology integration. The paucity of knowledge extends to nursing leadership capabilities that are emerging in response to the new leadership demands, including how nursing leaders can or should guide their teams within AI technology–enhanced environments. To date, nursing leadership theory and research have commonly focused on the tenets and processes of nurse leaders related to clinical workplace environments, empowerment, and care efficacy [5,6], without a specific investigation into the influence of AI technology on nursing leadership practice itself [7]. The limited focus on how AI technology has begun to evolve and reshape nurses’ roles within work environments highlights the growing knowledge gap in nursing leadership discourse.

Nurse leaders are essential to providing quality care [8-11], as evidenced by the association between relational leadership and associated patient outcomes. However, understanding how AI technology may potentially amplify nursing leadership to expand leadership capabilities is an appropriate next step for research inquiry. The existing body of nursing leadership literature has yet to fully consider or appreciate the potential for emergent AI technology to empower nurse leaders in a transformative way. As health care systems continue to adopt digital tools [12] and AI technology at a rapid pace, nurse leaders are uniquely positioned to guide ethical decision-making, advocate for equitable implementation, and model the integration of innovative tools that center on quality, safety, and compassion. However, the requisite support to empower these nurse leaders to succeed in AI technology–enhanced practices remains undefined.

The integration of AI technology into nursing workflows, policy, and care delivery may reshape the scope and focus of nurse leaders’ roles, particularly in relation to ethics, workforce management, education, and equity. There is a need to explore how nurse leaders are engaging with these shifts and to understand their perspectives, responsibilities, and strategic contributions within AI technology-augmented systems. While nurses are often viewed as end users or passive recipients of technological change [13], nurse leaders may have a critical role in shaping the ethical, operational, and relational dimensions of AI technology implementation when empowered to do so. As frontline innovators and stewards of care quality, nurse leaders are essential to ensuring that AI technologies align with professional values and serve diverse populations with integrity.

Having identified the number of gaps, synthesizing the current knowledge surrounding AI technology and nursing leadership is essential. Therefore, a scoping review methodology was selected to synthesize the existing research and discourse on nursing leadership and AI technology. Given the rapid pace of digital transformation in health care and the critical leadership role nurses play, a synthesis of the existing literature is needed to better understand how AI technology is intersecting with nursing leadership at present. This scoping review was designed to address that gap and provide a comprehensive overview of how nursing leadership is currently situated in relation to the rise of AI technology. Specifically, this review aimed to map the existing literature, identify key concepts and areas of focus, and identify key gaps to inform future research directions.

### Objective

This scoping review addressed the following question: What are the contemporary perspectives on AI technology and nursing leadership?

## Methods

### Overview

This scoping review applied the Joanna Briggs Institute (JBI) [14] scoping review framework methodology, which built upon the foundational work of Levac et al [15] and the 5-step methodology developed by Arksey and O’Malley [16]. The JBI framework included the following stages: (1) identifying the research question; (2) identifying relevant articles; (3) selecting articles; (4) charting the data; and (5) collating, summarizing, and reporting the results. The optional consultation exercise was excluded from this review [14]. The reporting for this scoping review followed the PRISMA-ScR (Preferred Reporting System for Systematic Review and Meta-Analyses extension for scoping reviews; Checklist 1) [17-20].

### Search Strategy

The authors collaboratively developed the search strategy using MeSH terms and text words for the population, concept, and context (PCC) framework related to the research question [14]: (1) nursing leadership, (2) AI, and (3) health care. The strategy was piloted to confirm the suitability of terms and was peer-reviewed by a research librarian using the Peer Review of Electronic Search Strategies checklist (Multimedia Appendix 1 provides detailed search string syntax) [21].

### Information Sources

The systematic search strategy conducted on May 21, 2024, targeted databases most relevant to the subject matter, including MEDLINE (Ovid), CINAHL, PsycINFO, and Scopus. Selected articles were manually reviewed for citations matching the inclusion criteria to identify additional articles not captured in the database searches, as well as Google (Google LLC) searches for gray literature. These approaches did not uncover any new articles that met the inclusion criteria.

### Eligibility Criteria

On the basis of the established PCC framework, inclusion and exclusion criteria were determined through an iterative team approach to establish the breadth and comprehensiveness of the scoping review [14,22]. For inclusion, articles had to explore the experiences and perspectives of nurse leaders and AI technology in health care, including or discussing actions undertaken by nurse leaders, with a focus on AI technology directly applicable to current nursing leadership practices in health care. Articles were excluded if they focused on AI technology in domains of nursing other than leadership, if they focused on nursing leadership but not AI technology, or if they mentioned nursing leadership only to a limited extent, with further exclusions applied to articles with outdated practices that lack alignment with current nursing professional practice measures.

**Figure 1.**
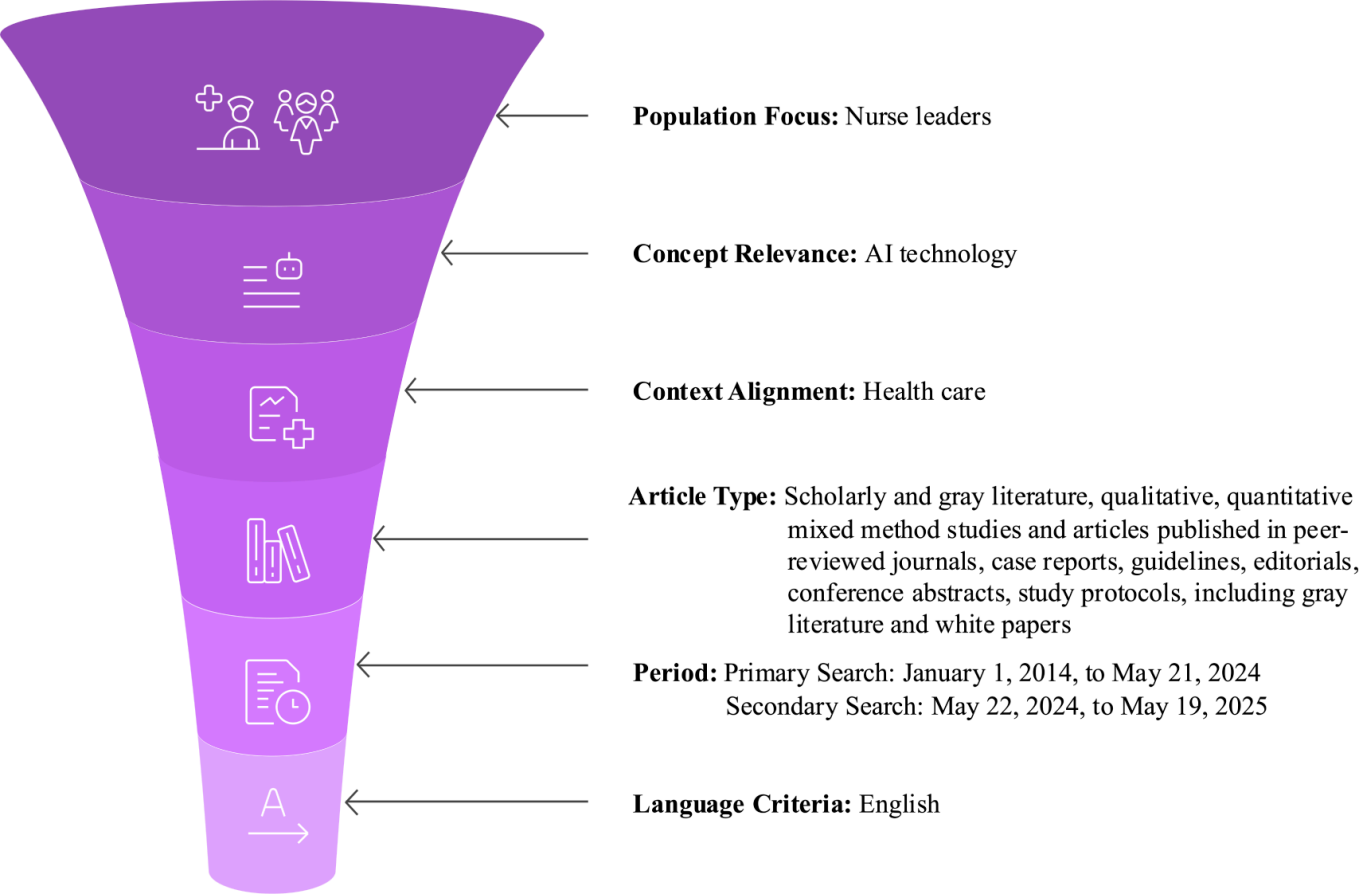
Funnel diagram illustrating the sequential application of key inclusion criteria for study selection.

The scope of evidence included qualitative, quantitative, or mixed methods studies or articles published in peer-reviewed journals, case reports, guidelines, editorials, conference abstracts, study protocols, and gray literature, including white papers and reports. All types of literature reviews, dissertations, books, and book chapters were excluded due to the scope of the project. Finally, only English-language articles were included due to limited translation resources. The primary search was limited to the most recent 10 years, specifically to articles published between January 1, 2014, and May 20, 2024, to capture the proliferation of contemporary AI technology in nursing leadership practice [23], ensuring a comprehensive review. The exact search process was replicated on May 19, 2025, to extend the scope of the review by 1 year in an attempt to provide updates to the review and reveal dynamic shifts in the subject matter. Finally, owing to limited translation resources, the language criterion was applied to align with the authors’ language fluency. The primary and supporting authors independently applied the inclusion and exclusion criteria to the retrieved articles from scholarly databases to assess the robustness of the criteria in capturing relevant publications. The application of the inclusion process is visualized in Figure 1, and all articles not meeting the inclusion criteria were excluded.

This figure visually maps the filtering process for sources of evidence, organized sequentially by the PCC framework [14]. The criteria were determined through an iterative team approach to establish the methodological boundaries of the scoping review [14,22]. The sequential process applies: (1) population focus (nurse leaders), (2) concept relevance (AI technology), and (3) context alignment (health care). Subsequent filters include article types and the specific period limits, which were set to capture the proliferation of contemporary AI technology in practice. The final step applies the language criteria (English) based on the fluency of the reviewers.

### Study Selection  

On retrieval of the search results from the scholarly databases, these results were uploaded to the web-based literature review platform software, Covidence (Veritas Health Innovation), to facilitate article screening and review. An example of the search results from both the primary and secondary searches from the Ovid MEDLINE database is available in Multimedia Appendix 2. The primary and secondary authors independently screened the titles and abstracts of retrieved articles against the inclusion and exclusion criteria using the Covidence platform. At this stage, articles that did not fulfill the inclusion criteria of the scoping review were excluded. Discrepancies from the screening of articles were resolved through discussion and consensus. Initially, 241 references were imported into Covidence for screening. After removing 46 duplicates, 195 articles were screened for title and abstract, and 85 articles were excluded. The remaining 110 articles were assessed for full-text eligibility against the inclusion and exclusion criteria, resulting in the exclusion of 96 articles and the inclusion of 14 articles in the final extraction. As of May 2024, 14 articles were included. Following an updated search and review process in May 2025, using the same search protocol with articles published from May 2024 to May 2025, 124 new articles were uploaded for screening into Covidence. The 124 articles were automatically reduced to 101 for screening by removing duplicates. The abstract and title screening process resulted in the exclusion of 57 articles, with 44 articles retrieved for full-text eligibility. This process identified an additional 12 articles, which were included in the final review. Combined with the initial 14 articles, the extended search of an extra 12-month period has resulted in a total of 26 articles included in the final data extraction and synthesis phase.

### Data Extraction

The data charting process was facilitated by an adapted JBI extraction tool (Joanna Briggs Institute) [14] via an Excel (Microsoft) spreadsheet, which was used to collect, transcribe, and organize data aligned with the core descriptive elements related to the research question. Data captured during the manual extraction process was transcribed into an Excel spreadsheet in the following categories: authors, publication year, country, design, aim, AI technology context and type, and findings [14,18]. The authors reviewed and piloted the adapted extraction tool on 5 articles before the full data collection process to ensure standardized extraction. The piloting process was assessed as successful, and it was determined that no changes to the adapted extraction tool or data collection approach were necessary.

### Critical Appraisal of Individual Sources of Evidence

Owing to the nature of scoping reviews and the intention of providing an overview of existing literature (rather than an assessment of rigor or quality), the articles were not evaluated for risk of bias or methodological quality [20].

### Data Analysis

This scoping review followed guidance from the JBI to conduct an inductive qualitative content analysis and numerical summary to establish key data categories, trends, and identifiable gaps in the literature to summarize the evidence and report information to map the literature on this evolving topic [14,18,19,24,25]. The iterative analysis process ensured a rigorous and transparent extraction of content on the intersection of nursing leadership and AI technology using a standardized data extraction sheet informed by the JBI methodology [14].

A basic qualitative content analysis conducted by the primary author facilitated the categorization of the extracted data [18]. The analysis process began with familiarization with the data through repeated readings of the evidence sources, followed by open coding to extract relevant information and establish coding categories [26]. The data extracted from the 14 documents were then organized into a hierarchical structure that facilitated the active emergence of 6 overarching categories of data to address the phenomenon of the intersection of nursing leadership and AI technology in a conceptual form [26] (Multimedia Appendix 3). These categories were then analyzed numerically by counting the frequency at which each data category actively emerged [26]. This same rigorous approach to data analysis was applied to 12 articles that met the inclusion criteria, retrieved in May 2025 through a secondary search conducted a year later, and updates to the primary results were included to extend the value of the findings.

## Results

### Study Characteristics

**Figure 2.**
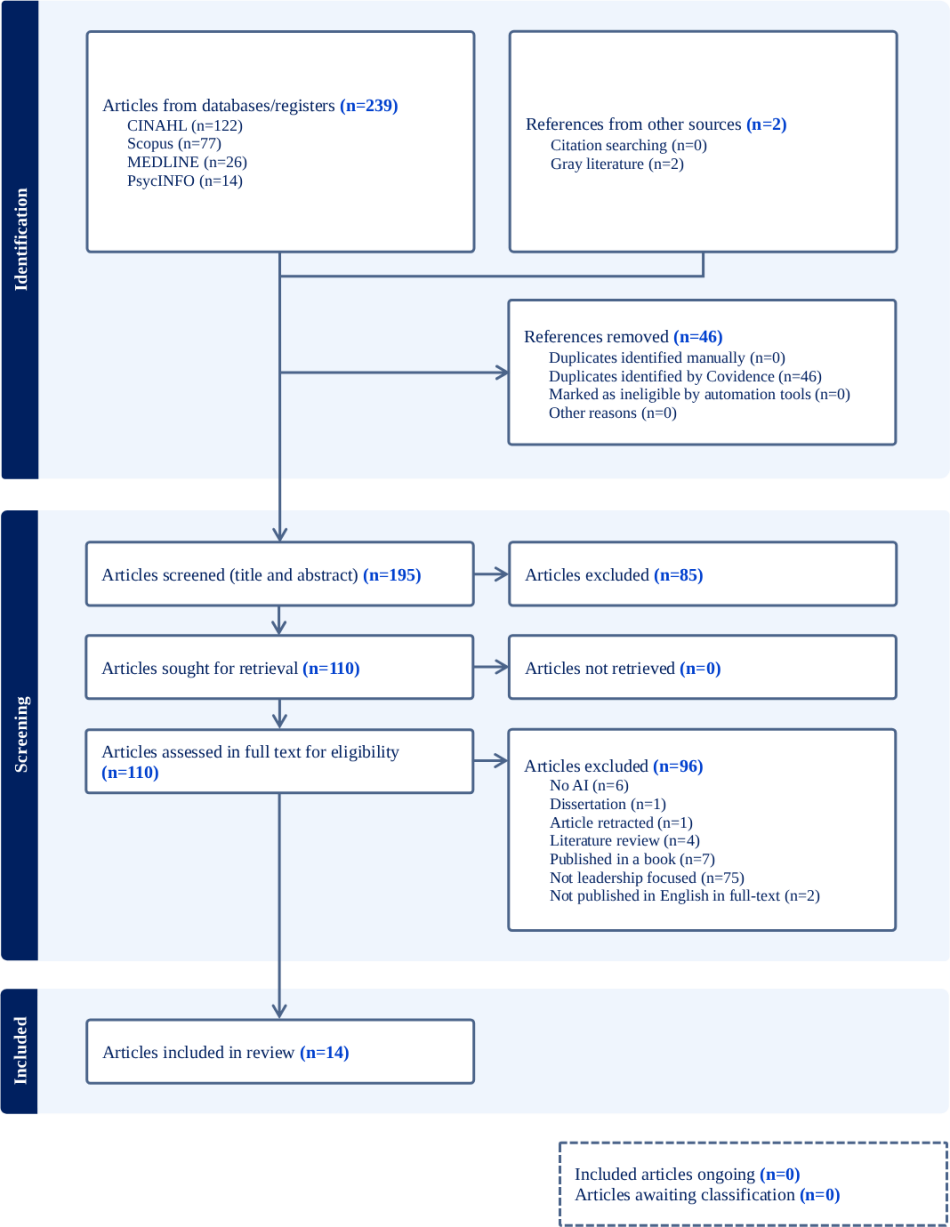
PRISMA-ScR (Preferred Reporting Items for Systematic Reviews and Meta-Analyses Extension for Scoping Reviews) flow diagram (primary search): This figure promotes transparency and reproducibility by mapping the 4 key stages (identification, screening, eligibility, and inclusion) for the initial 195 screened articles (January 1, 2024, to May 21, 2024), resulting in the final 14 included articles (14/195, 7.2%).

**Figure 3.**
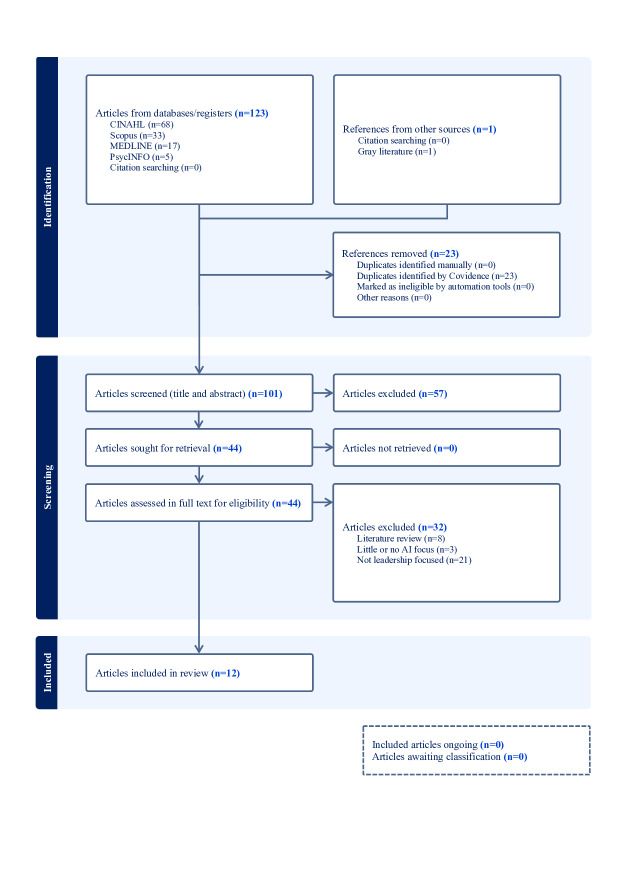
PRISMA-ScR (Preferred Reporting Items for Systematic Reviews and Meta-Analyses Extension for Scoping Reviews) flow diagram (secondary search): This figure details the organized selection process through the 4 key stages (identification, screening, eligibility, and inclusion) for the extended search (May 22, 2024, to May 19, 2025), mapping the 101 screened articles that yielded 12 (12/101, 11.9%) additional included articles.

**Figure 4.**
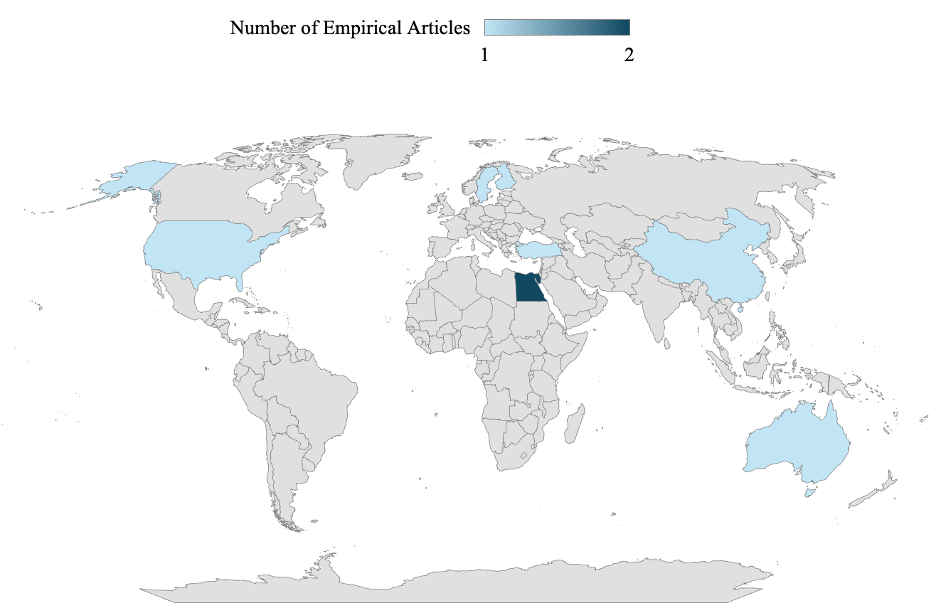
Empirical articles (n=8) mapped by country (Australia, n=1, 12.5%; China, n=1, 12.5%; Egypt, n=2, 25%; Finland, n=1, 12.5%; Sweden, n=1, 12.5%; Turkey, n=1, 12.5%; and the United States, n=1, 12.5%).

**Figure 5.**
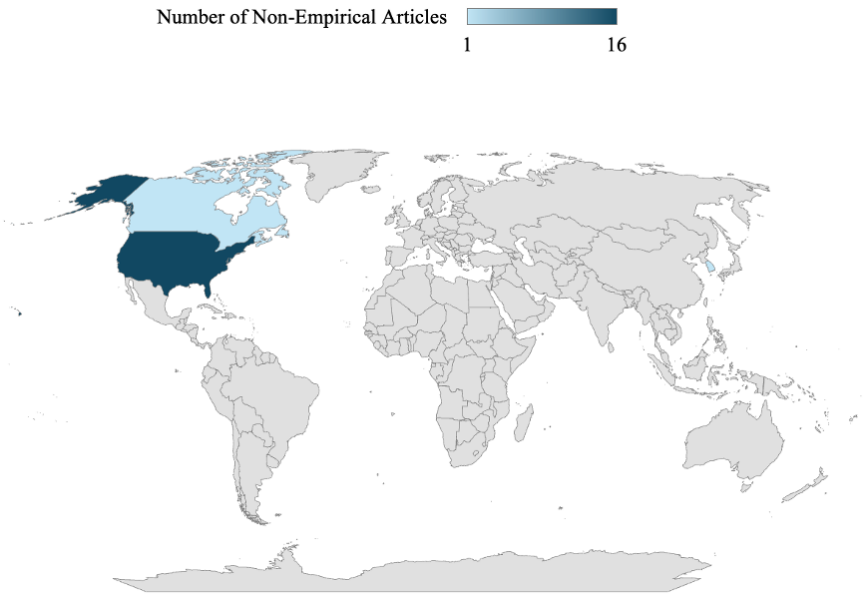
Nonempirical articles (n=18) mapped by country (the United States, n=16, 88.9%; Canada, n=1, 5.6%; and the Republic of Korea, n=1, 5.6%).

The final review included 26 articles published between 2014 and 2025, with a marked increase in empirical studies in 2024 and 2025. In the primary search, 195 articles were identified, of which 14 (14/195, 7.2%) met the inclusion criteria (Figure 2). Of these, 11 (11/14, 78.6%) were commentaries, editorials, or perspectives [12,27-36] published in the USA, and 1 (1/14, 7.1%) empirical article each came from Turkey [37], Finland [38], and China [39]. In the secondary search, 124 articles were identified, of which 12 (12/124, 9.7%) met the inclusion criteria, resulting in a total of 26 articles included in the review (Figure 3). The secondary search results indicate an increase in empirical research, as reflected in 5 of the 12 articles included [40-44], which represent singular empirical articles from Sweden, Australia, and the United States and 2 from Egypt (Figure 4 maps the empirical articles by country). The remaining 7 articles were expert or professional commentaries, a theoretical paper, and a quality improvement study [45-51] representing single articles from Canada and the Republic of Korea and 5 from the United States (Figure 5 maps the nonempirical articles by country). Of the 26 included articles, 8 are empirical (qualitative, quantitative, or mixed methods), and 18 were expert commentaries, perspectives, editorial, or theoretical articles

### Content Analysis Findings

#### Overview

The qualitative content analysis of the primary and secondary searches revealed 6 overarching data categories that actively emerged through the inductive and rigorous analysis approach: (1) leading digital transformation and technology integration, (2) AI technology and the nursing role: reshaping practice, (3) ethical considerations of AI technology for nurse leaders, (4) AI technology as a facilitator of innovative leadership, (5) education and training on AI technology in nursing practice, and (6) influence of AI technology on the work environment. This section relates the mapped evidence relating to the 6 data categories, focusing on the numerical analysis that details the number of articles included in each category, as well as the character framework that provides a contextual map of the evidence. The synthesis and interpretive commentary are provided in the discussion.

#### Leading Digital Transformation and Technology Integration

The intersection of nursing leadership and AI technology is described in the context of digital transformation and technology integration in 10 of 14 (71.4%) articles from the primary search [12,27,28,30,34-39] and 10 of 12 (83.3%) articles from the secondary search [41-47,49-51] for a total of 20 of 26 (76.9%).

In the primary search, most contributions were commentary or perspective articles, with limited empirical evidence. Laukka et al [38] reported that Finnish nurse leaders guided the implementation of AI while addressing patient safety and staff concerns. Li et al [39] surveyed 263 Chinese nurses and found that nurse leaders were expected to lead the integration of technology and involve staff in decision-making. Eminoğlu and Çelikkanat [37] studied 196 Turkish nurse leaders and found a positive association between leadership self-efficacy and AI technology readiness.

Commentaries emphasized nurse leadership as a key factor in digital transformation. Porter-O’Grady [34] advised nurse leaders to support value-based care during digital change, Blouin [27] suggested frameworks with evaluation metrics, and Linnen et al [12] promoted AI technology adoption to enhance patient safety and reduce hospital-acquired outcomes. Cineas et al [28] argued that nurse-led AI technology initiatives can promote equity in care delivery through designs that incorporate social determinants of health. Siedlecki [35] reported that AI technology can amplify nursing research through transformative approaches, associated with cautioning about ethical risks in digital environments.

In the secondary search, empirical evidence was more prominent. Sloss et al [49] conducted a mixed methods study of 120 US nurse leaders and reported that structured governance processes were associated with greater confidence in using AI technology to enhance patient safety and equity. Kotp et al [42] surveyed 187 Egyptian nurse leaders and found that one-third reported high readiness for AI-driven predictive analytics, with readiness influenced by age, education, and employment status. Lundsten et al [43] surveyed 142 US nurse leaders and reported that organizational support and resources predicted higher engagement in digital transformation. Neves et al [44] interviewed 18 stakeholders in the long-term care sector in the United Kingdom and reported both optimism about AI technology and concerns regarding trust and responsibility. Finkelstein et al [41] studied perioperative staff in Sweden and emphasized the need to align AI technology with workflows and staff expectations.

Commentary and perspective articles reinforced similar themes, including those described by Cardiff et al [46], who noted that nurse executives are integral to shaping AI technology strategies and governance. Hoelscher et al [47] emphasized the need for AI technology skills to implement systems responsibly and safely. Aldrich et al [45] positioned AI technology as an extension of nursing’s mission. Tyransky et al [50] and Virkstis [51] described executives and emerging leaders preparing for AI technology adoption through mentorship and innovation.

#### AI Technology and the Nursing Role: Reshaping Practice 

Most articles included in both the primary (11/14, 78.6%; [12,27,28,30-34,37-39]) and secondary (8/12, 66.7%; [41,42,44-47,50,51]) reviews described how AI technology was impacting aspects of the nursing role, accounting for 19(73.1%) of the 26 included articles.

Sullivan et al [36] described a proactive approach to integrating AI technology into nursing practice, reporting its use to support leadership functions, such as resource allocation and predictive analytics, to inform patient care strategies and staffing. AI technology was also reported to alleviate administrative burdens, affording nurse leaders time to focus on strategic leadership [36]. This proactive approach aligns with Blouin [27] and Clipper et al [30], who described how nurse leaders can take an active role in directing how AI technology augments nursing work by shifting attention away from non–value-added tasks and back to direct patient care. Fuller and Hansen [33] emphasized the importance of including nurses as leaders of digital adaptation through participatory processes in AI technology implementation, with active inclusion in decision-making described as promoting ownership in reemphasized nursing roles. Similarly, Douglas and Gray [31] described the need for strategic approaches, involving the urgency for nurse leaders to adapt to the changing AI landscape and to support shifting generational expectations in practice.

AI technology was also described as a potential disruptor with the capacity to reshape roles in pursuit of equity. Cineas et al [28] and Linnen et al [12] described nurse-led AI technology as an opportunity to reduce task burden, empower nurses, and redirect their energy toward meaningful engagement. Porter-O’Grady [34] emphasized that digital leadership could promote equity and gender parity in a profession navigating entrenched hierarchies, but that this required reshaping roles to encourage the active engagement of nurses as technology leaders. Laukka et al [38] reported that team engagement with AI technology can improve workflows and increase patient interaction, whereas Li et al [39] emphasized that role reshaping must be actively led by nurse leaders who intentionally involve staff in decisions at all stages. Eminoğlu and Çelikkanat [37] reported on the relationship between leadership self-efficacy and readiness for AI, underscoring the link between leadership capacity and role expectations in technology adoption.

Another element described was the need to redefine the nursing role through the development of expertise in research and data science. Linnen et al [12] emphasized the importance of graduate education in building digital competencies and mitigating the scarcity of nurse scientists with expertise in AI and data science. Laukka et al [38] similarly called for more nurse-led AI research. Fontenot [32] described the importance of integrating AI technology into nursing education to equip nurses with the necessary skills to guide digital advancements and data-driven care delivery.

Articles from the secondary search further expanded these findings. Aldrich et al [45] described how predictive analytics were shifting nurse-led decision-making and care planning. Cardiff et al [46] emphasized the need to align AI technology innovation with nursing values, ensuring that changes in practice support the human presence at the heart of care. Finkelstein et al [41] reported that integration of AI technology into leadership thinking prompted new ways of working, with nurses expected to navigate both clinical and digital domains. Hoelscher et al [47] described how AI technology could reduce administrative tasks while creating expectations for enhanced data interpretation and system navigation. Kotp et al [42] reported that AI-enabled decision support reshaped documentation and altered how nurses prioritized clinical cues. Neves et al [44] highlighted how AI technology in care homes was influencing delivery and relational dynamics, prompting staff to reevaluate trust and role expectations. Tyransky et al [50] described how leaders were considering changes to team roles and structures to preserve the relational aspects of nursing as AI technology entered workplaces. Virkstis [51] reported that executives were reimagining workflows and competencies needed to support nursing roles in increasingly digital environments. Across these articles, AI technology was consistently described not as replacing nursing but as reshaping how nurses think, interact, and lead within a changing clinical landscape.

#### Ethical Considerations of AI Technology for Nurse Leaders 

A total of 12 (12/26, 46.2%) articles, including five (5/12, 41.6%) from the primary search [27,28,32,35,36] and seven (7/12, 58.3%) from the secondary search [42,44-47,49,51], directly addressed the ethical implications for nurse leaders regarding the increasing use of AI technology in nursing.

Cineas et al [28] described nurse-led AI technology initiatives as central to promoting health equity by supporting ethical care and mitigating bias in patient care. Siedlecki [35] emphasized both the potential benefits of AI technology, such as enhancing nursing research, and the risks associated with data breaches and inaccuracies. Fontenot [32] highlighted the importance of ethical considerations and patient safety when implementing AI technology, noting that AI can help close the research-to-practice gap but must be guided by principles of equity and ethics. Blouin [27] stressed the importance of evaluating and reassessing AI technologies to mitigate ethical risks to autonomy and decision-making. Sullivan et al [36] described how AI technology adoption creates new leadership responsibilities by introducing ethical challenges while offering opportunities to empower teams.

Articles from the secondary search further underscored ethical themes. Cardiff et al [46] emphasized that ethical decision-making cannot be delegated to algorithms, urging nurse leaders to remain grounded in values of dignity, presence, and justice. Sloss et al [49] reported on the importance of governance structures, describing how nurse leaders must be actively engaged in AI-related decision-making to protect patient safety and equity. Kotp et al [42] addressed automation bias and the unintended consequences of overreliance on AI technology decision support, emphasizing the leader’s role in defining safe boundaries of use. Hoelscher et al [47] described the responsibility of nurse leaders to preserve the mission of nursing by ensuring that AI technology tools support relational care and advocacy. Aldrich et al [45] noted that ethical awareness must be embedded within AI technology adoption strategies, particularly when predictive tools guide clinical decision-making. Neves et al [44] reported on long-term care settings, where AI technology created uncertainty around responsibility and judgment, highlighting the need for leadership to safeguard ethical clarity and trust. Virkstis [51] emphasized the need for transparency and accountability, reporting that nurse executives play a central role in building frameworks to guide teams through ethical challenges. Across these articles, ethical AI technology leadership was consistently described as an active responsibility for nurse leaders and an essential component of successful AI technology integration.

#### AI Technology as a Facilitator of Innovative Leadership

A frequent theme identified in 9 (64.3%) of 14 articles from the primary search [12,29,31-34,36,37,39] and in 6 (50%) of 12 articles from the secondary search [42,45-47,49,51], for a total of 15 (57.7%) of 26 included articles, was the potential for nurse leaders to harness AI technology as a facilitator of nursing leadership through innovative practices.

Porter-O’Grady [34] described innovative leadership practices in which nurse leaders act as change agents by embracing digital technology as a tool to transform practice. Fuller and Hansen [33] reported on nurse leaders as disruptors who guided the integration of AI technology to support evidence-based nursing. Clancy [29] noted that innovative approaches could help mitigate workforce shortages by leveraging AI technology to apply lean principles such as waste reduction and value creation. Linnen et al [12] described AI technology integration as an opportunity for nurse leaders to dismantle external limitations on the profession and prepare digitally competent nurses. Sullivan et al [36] emphasized a balanced, innovative stance toward AI technology, noting its potential to improve leadership practices while extending benefits to teams through empowerment and cautious discretion.

Additional primary search contributions included Li et al [39], who found that innovative leadership behaviors increased staff enthusiasm for innovation. Douglas and Gray [31] emphasized the need for adaptive leadership approaches to address generational shifts in the workforce. Fontenot [32] described how innovative leadership within AI-augmented environments required balancing evidence-based practice with ethical considerations to safeguard patient safety. Eminoğlu and Çelikkanat [37] conducted a descriptive correlational study involving 196 nurse leaders in Turkey, reporting a positive association between medical AI technology readiness and leadership self-efficacy, underscoring the link between readiness and leadership development.

Articles from the secondary search further reinforced AI technology as a catalyst for innovative leadership. Aldrich et al [45] described how nurse leaders aligned AI technology adoption with strategic foresight to improve care planning and operational efficiency. Cardiff et al [46] emphasized agile and responsive leadership models that integrate AI technology while maintaining human values. Hoelscher et al [47] identified opportunities for AI technology to reinforce nursing’s mission through transformative innovation, benefitting patients, teams, and systems. Kotp et al [42] highlighted how creativity and flexibility in leadership supported successful implementation and sustained innovation. Sloss et al [49] reported that participation in structured governance processes increased leaders’ confidence in guiding innovation responsibly. Tyransky et al [50] described how AI technology was shifting leadership structures and inviting new forms of influence. Virkstis [51] emphasized how executives engaged in systems-level innovation, framing AI technology as an opportunity to elevate nursing leadership and drive meaningful transformation. Across these articles, nurse leaders were consistently portrayed as visionaries and architects of change, actively shaping how AI technology was introduced, adapted, and sustained in practice.

#### Education and Training on AI Technology in Nursing Practice

The primary search identified 10 articles (10/14, 71.4%) [12,27,28,30,31,33-36,38,39], and the secondary search identified 5 (5/12, 41.7%) additional articles [42,46,49-51], for a total of 15 (57.7%) of 26 articles that focused on education and training in AI technology for nursing practice. These articles addressed both the implementation of training for staff nurses and the development of AI technology expertise for nurse leaders.

Blouin [27], Porter-O’Grady [34], and Laukka et al [38] described the importance of cultivating a culture of continuous learning to ensure staff readiness for digital tools and maintain relevance in evolving health care environments. Siedlecki [35] highlighted the benefits of AI technology in research and emphasized the importance of ongoing digital education, focusing on ethical use, data privacy, and security. Douglas and Gray [31] emphasized tailoring education to generational learning needs, describing adaptive approaches to address workforce complexity. Linnen et al [12] advocated for formal digital education at the doctoral level, including funding and institutional support to prepare nurse scientists for leadership in a physician-dominated sector.

Several authors [28,30,33,36] have described the need for education that spans the full AI technology lifecycle, from development through implementation, use, and evaluation, to position nurses as leaders and embed AI technology into professional development strategies. Li et al [39] reported that nurse leaders who facilitated digital education for teams promoted positive psychological capital through enhanced self-efficacy.

Articles from the secondary search further emphasized education as a core consideration for nursing leadership. Kotp et al [42] reported on the relationship between leadership readiness and digital literacy, recommending ongoing education to support meaningful interpretation of predictive analytics. Cardiff et al [46] described the need to build digital confidence by normalizing iterative learning and providing practice-based training. Sloss et al [49] underscored the importance of preparing nurses for governance roles, highlighting that meaningful involvement in AI technology decision-making depends on adequate education. Tyransky et al [50] reported that think tanks identified professional development, mentorship, and accessible education as key enablers of innovative leadership. Virkstis [51] described a national initiative that empowered emerging nurse informaticians through structured education and exposure to implementation science, demonstrating how training builds leadership capacity in digital transformation. Across these articles, education was consistently framed not as a 1-time requirement but as a continuous, strategic investment to prepare and sustain nurse leader expertise for AI-enabled health care.

#### Influence of AI Technology on the Work Environment

The influence of AI technology on the work environment from a nurse leadership perspective was described in 7 (50%) of 14 articles from the primary search [12,27,29,30,34,36,39] and 5 (41.7%) of 12 articles from the secondary search [41,42,44,49,50], totaling 12 (46.2%) of 26 included articles.

In the primary search, Blouin [27] described the strategic implementation of AI technology to reduce workload and administrative burden, reporting that automation of repetitive tasks enabled nurses to focus on patient care. Sullivan et al [36] reported that AI technology could help address workforce shortages by predicting patient needs and guiding staffing allocation. Clancy [29] emphasized AI technology as a tool to improve workflows and mitigate the impact of shortages. Clipper et al [30] described the use of AI technology to reduce nonpatient tasks and enhance collaboration by involving nurses in system design. Linnen et al [12] described AI technology as an opportunity to disrupt structures that perpetuate nurse oppression, positioning nurse-led technology as a means to create empowerment. Li et al [39] reported that involving staff in shaping how AI technology is used promoted job control and more equitable work environments.

In the secondary search, Finkelstein et al [41] conducted a qualitative study in Sweden with perioperative staff, including nurses, managers, and physicians, examining expectations of an AI technology tool predicting length of stay, reporting that alignment with workflows and user trust were key to successful integration. Kotp et al [42] described predictive analytics as reducing administrative burden and enabling more strategic leadership presence. Neves et al [44] reported that AI in long-term care shifted interpersonal dynamics and caregiving models, requiring leadership to preserve relational care. Sloss et al [49] emphasized that equitable governance in AI implementation directly impacted workplace climate and professional autonomy. Tyransky et al [50] described perspectives from leadership think tanks, noting AI’s potential to optimize staffing and reduce burnout if aligned with nursing values and frontline involvement. Across these articles, nurse leaders were consistently positioned as pivotal to shaping AI-informed work environments that are efficient, ethical, and grounded in professional nursing practice.

## Discussion

### Principal Findings

This scoping review confirms the critical role of nursing leadership in AI technology integration, despite a primary finding being the profound scarcity of empirically validated knowledge in this emergent area. Of the 26 included articles, the vast majority (18 [69.2%] were expert commentaries, perspectives, editorials, or theoretical articles) significantly outweighed the 8 [30.8%] empirical studies (qualitative, quantitative, or mixed methods studies) identified. The considerable imbalance indicates that existing research is predominantly theoretical, revealing an important research gap in knowledge that needs to be addressed to inform the development of evidence-based, actionable strategies. The inclusion of recent empirical studies demonstrates a shift from primarily theoretical discourse toward actionable strategies, readiness assessments, and program evaluations. Nurse executives emerged as key actors in policy, governance, and implementation, underscoring the importance of nursing leadership voices in shaping the adoption of AI technology. The updated dataset further revealed a nuanced understanding of nurse leaders as change agents, ethics stewards, and education champions. However, variability in AI technology literacy and readiness underscored the need for strategic training and system-level support.

### Leading Digital Transformation and Technology Integration

Findings confirmed the positioning of nurse leaders as central to envisioning, guiding, and supporting AI-related shifts in care delivery, operations, and workforce planning [12,27,28,30,34-39,41-47,49-51]. Nurse leaders were described as innovators balancing technological adoption with professional values, ensuring alignment between digital transformation and nursing practice. Empirical studies have shown that organizational support, governance structures, and leadership readiness significantly influence successful AI technology adoption [38,42,43,49]. Interpretations suggest that sustained investment in leadership development is necessary to prepare leaders for expanded digital governance responsibilities.

### AI Technology and the Nursing Role: Reshaping Practice

The review highlighted that AI technology is reshaping nursing roles rather than replacing them [12,27,28,30-34,37-39,41,42,44-47,50,51]. Leaders who involved staff in AI technology decisions fostered job control, equity, and professional empowerment [38,39]. Education was framed as essential to redefining the role, particularly through graduate preparation in digital skills and nurse-led research [12,38]. Interpretively, these findings suggest that nurse leaders must intentionally engage staff to transform potential disruption into empowerment and equity.

### Ethical Considerations of AI Technology for Nurse Leaders

AI technology was positioned as both a potential benefit and a source of ethical risk [27,28,32,35,36,42,44-47,49,51]. Leaders were described as active agents in safeguarding dignity, presence, and justice while mitigating risks such as automation bias, privacy concerns, and inequities as AI technology shifts the health care landscape. Empirical and commentary articles emphasized the need for transparent governance, ethical frameworks, and transparency in decision-making. Interpretively, ethical nursing leadership in the integration of AI technology must move beyond passive oversight to proactive engagement, embedding ethical awareness into governance structures and daily practice.

### AI Technology as a Facilitator of Innovative Leadership

Nurse leaders were portrayed as visionaries and architects of change, using AI technology to drive innovative leadership practices [12,29,31-34,36,37,39,42,45-47,49,51]. Empirical work supported associations between leadership self-efficacy and AI technology readiness [37], whereas commentary articles emphasized opportunities for agile and responsive leadership [45-47]. Collectively, the literature frames AI technology not simply as a tool, but as a catalyst for leadership innovation at both the organizational and system levels. Interpretively, this highlights the urgent need for nurse leaders to intentionally develop competencies in digital literacy, relational leadership, and co-creation with multidisciplinary partners.

### Education and Training on AI Technology in Nursing Practice

Education emerged as a central leadership responsibility in both primary and secondary education searches [12,27,28,30,31,33-36,38,39,42,46,49-51]. Authors described the importance of fostering continuous learning cultures, tailoring education to generational needs, and preparing doctoral-level nurse scientists. Empirical studies emphasized the relationship between leadership readiness and digital literacy [42]. These findings suggest that education should be viewed as a continuous, strategic investment to sustain nurse leader acumen in leading the integration of AI technology and empowering their teams to enhance care delivery through digital transformation.

### Influence of AI Technology on the Work Environment

The influence of AI technology on the work environment was a central topic in leadership discussions [12,27,29,30,34,36,39,41,42,44,49,50]. Leaders were described as pivotal in leveraging AI technology to reduce administrative burden, improve workflow efficiency, and mitigate burnout [27,29,30,36,42,50]. Empirical findings underscore the importance of aligning AI technology systems with existing workflows and preserving trust and relational care [41,44]. Interpretively, these results highlight AI technology as both a facilitator of efficiency and a disruptor of traditional care models, requiring intentional leadership to ensure adoption aligns with nursing values and professional autonomy. AI technology, leveraged by nurse leaders, also presents an opportunity to change systems that impact nurses, transforming the work environment into one that benefits both nurses and patient care. Substantive opportunities to leverage AI technology are appropriately guided by informed nurse leaders who involve staff in decisions related to AI technology. Li et al [39] specifically found that leaders who intentionally facilitate work environment strategies can mitigate burnout and improve job satisfaction during AI integration. Conversely, the potential risks of failing to engage with AI technology may result in missed opportunities to enhance nursing practice, patient care delivery, and the work environment. Taken together, these findings demonstrate that the adoption of AI technology reshapes the work environment and demands leadership presence in guiding organizational change.

### Research Gaps, Global Perspectives, and the Canadian Context

The findings of this scoping review suggest that effective nursing leadership plays a crucial role in integrating AI technology within health care systems. The inclusion of recent empirical work expanded global perspectives from Egypt, Sweden, and Korea, enriching applied knowledge of actionable leadership strategies, readiness assessments, and program evaluations [40-43,47]. The updated dataset reinforces the prominent role of nurse executives as change agents, ethics stewards, and education champions [41-44,46,49-51], yet findings reveal persistent variability in AI technology literacy and readiness, highlighting a continuous need for strategic training and system-level support.

As AI technology continues to instigate change in health care delivery, advancements are rapidly shifting nursing practice, and the requisite expertise of nurse leaders is outpacing research.

The lack of scientific inquiry at the intersection of nursing leadership and AI technology is evident in the profound scarcity of empirical research currently available. Of the 14 articles from the primary search, only 3 were empirical studies, all conducted outside of North America [37-39]. The paucity of empirical inquiry highlights a global gap, marked by a notable absence of a Canadian presence. Although the secondary search added 5 empirical studies [40-44] within a 12-month period, a significant increase when compared to the previous decade, Canadian representation in empirical research remains absent, despite evidence that Canadian nurse leaders are already engaging with AI technology in practice [4]. This gap highlights the urgency for targeted funding, infrastructure, and academic partnerships to support Canadian nurse scientists and leaders in AI technology research [52]. The sharp increase in publications during the secondary search period also signals growing global attention and a time-sensitive opportunity for nursing leadership to claim space in AI development.

A Canadian presence within the AI technology and nursing leadership research space is crucial to match the nuanced landscape of our health system and diverse patient needs [52]. The remaining articles included for review (ie, commentaries and editorials authored in the USA) reflect the intense interest of nurse leaders as the arena continues to change shape under the influence of AI technology. As nursing leadership demands expand with AI technology, creating a new leadership practice dimension, nurse leaders need not only to shift to adapt but also take a definitive role in shaping how AI technology influences practice [27,28,30,32-34].

Together, these articles highlight the multifaceted nature of nursing leadership in the era of AI technology, emphasizing the profession’s dual role as both caregivers and architects of transformation. The findings affirm that intentional leadership development, ethical discernment, and education grounded in the realities of nursing work are essential to guiding this integration in ways that honor both technological advancements and the core values of the profession, which may have a profound impact on our profession moving forward.

### Limitations

This review has limitations that need to be described. First, while every attempt was made to include articles relevant to the topic, because of the specificity of the inclusion criteria, only 14 articles that met the eligibility criteria were identified in the initial search, with a further 12 added through the secondary extended timeline search. In addition, despite a broad search strategy to ensure a wide capture of relevant information reflective of the scoping review process, limitations based on the inclusion and exclusion criteria may have limited the findings. These limitations include the language of publication being restricted to the English language and the exclusion of dissertations and book chapters due to the capacity of the inquiry team. Although the publication date range for the initial search was set at 10 years, this restriction likely did not limit the results, as the relevant articles matching the inclusion criteria were published from 2018 onward. Interestingly, when the publication date range was extended by 1 year for the secondary search, the number of included articles from a 1-year period, 12 articles, almost matches the 14 originally included from the previous 10-year period. This significant increase in included articles, especially empirical studies, which were noted to be only three in the previous decade, reveals the increase in research and interest in this subject matter.

Despite a broadened data set, limitations remain. Language was restricted to English due to resource constraints, potentially excluding relevant non-English articles. The review included theoretical and commentary-based articles, which, while valuable, may lack empirical validation. The diversity of AI applications and leadership roles made the standardization of synthesis challenging. Nonetheless, the expansion from 14 to 26 articles improved representativeness and thematic saturation.

### Conclusions

This scoping review provided insights into the contemporary perspectives on AI technology and nursing leadership, resulting in the identification of a significant research gap and a lack of scientific inquiry in this emergent area of leadership practice, which, with the secondary search, reveals promising signs of advancement in empirical research. The demand for leadership discussion and development is an essential area for advancing the profession, as it enables the maintenance of parallel expertise with the growth of this leadership dimension [31,33,34,36]. Furthermore, this investigation into nursing leadership and AI technology revealed a unique opportunity for nurse leaders to promote health equity in care delivery and professional equity for nursing itself, with the potential to alleviate oppressive practices that have hindered the actualization of the profession [28,34].

Future research should prioritize inclusive, empirical explorations that elevate diverse leadership voices and deepen understanding of AI technology’s transformative role in nursing leadership practice, with particular attention to the work environment. Future research should use diverse methodologies, including longitudinal and mixed methods designs, to examine how nurse leaders engage with AI technology across varying contexts. Comparative and interprofessional studies, as well as inquiry focused on rural, Indigenous, and underserved populations, are needed to inform both leadership practice and policy frameworks.

Positioning nurses as AI technology directors presents an opportunity for the nursing profession to influence and define its own future, rather than being influenced by external forces.

Douglas and Gray [31] emphasize the need for tailored AI technology education approaches to support nurses in thriving in practice. Specific nurse leadership education for practicing nurse leaders should be developed in tandem by nurse scientists and leaders to promote and support nurse leaders as change agents in the integration of AI technology into practice. Taken collectively, it is clear that the nursing profession needs to become fully engaged in the rapidly evolving landscape of AI technology [30,36,37].

## Supplementary material

10.2196/80085 Multimedia Appendix 1 Literature review detailed search string syntax.

10.2196/80085 Multimedia Appendix 2 Literature review search strategy results.

10.2196/80085 Multimedia Appendix 3 Overview of findings.

10.2196/80085 Checklist 1 Preferred Reporting Items for Systematic Reviews and Meta-Analyses extension for Scoping Reviews (PRISMA-ScR) checklist.

## Abbreviations

AI: artificial intelligence
JBI: Joanna Briggs Institute
PCC: population, concept, and context
PRISMA-ScR: Preferred Reporting Items for Systematic Reviews and Meta-Analyses Extension for Scoping Reviews
