# Supplementary material for: Exploring the Intersection of Nursing Leadership and Artificial Intelligence: Scoping Review
Source: JMIR Nurs. 2025 Nov 14;8:e80085. doi: 10.2196/80085 (PMC12617831; doi:10.2196/80085)
Supplement: Multimedia Appendix 1 [file nursing-v8-e80085-s001.pdf]

Multimedia Appendix 1

Literature Review Detailed Search String Syntax:

"Nursing administration" or "Nursing management" or "Leadership in nursing" or "Clinical leadership (in a nursing context)" or "Nursing directors" or "Nursing executives" or "Healthcare leadership (with a focus on nursing)" or "Nurse leadership roles" or "Leadership roles in nursing" or "Nursing supervisors" or "Nursing governance" or "Senior nursing professionals" or "Nurse managers" or "Chief Nursing Officer (CNO) and other executive roles" or "Nursing headship" or "Nursing command" or "Nursing chiefs" or "Nursing officer roles" or "Professional leadership in nursing" or "Charge Nurse" or "Head nurse" or "Nurse leader" or "Nursing leadership"
AND
"Artificial intelligence" or "AI" or "Machine intelligence" or "Computational intelligence" or "Decision-support systems" or "Automated decision-making" or "Intelligent systems" or "Smart systems" or "AI technology" or "Intelligent automation" or "Robotics and AI" or "Machine learning" or "Deep learning" or "Neural networks" or "Algorithmic intelligence" or "Cognitive computing" or "AI applications" or "AI tools" or "AI solutions" or "Advanced analytics" or "Data science" or "Predictive analytics"
AND
"Health care" or "Healthcare" or "Medical care" or "Clinical care" or "Patient care" or "Health services" or "Medical services" or "Clinical services" or "Health systems" or "Medical systems" or "Clinical systems" or "Health management" or "Medical management" or "Clinical management" or "Health service delivery" or "Medical service delivery" or "Clinical service delivery" or "Healthcare sector" or "Medical industry" or "Health industry"
