# Supplementary material for: Exploring the Intersection of Nursing Leadership and Artificial Intelligence: Scoping Review
Source: JMIR Nurs. 2025 Nov 14;8:e80085. doi: 10.2196/80085 (PMC12617831; doi:10.2196/80085)
Supplement: Multimedia Appendix 2 [file nursing-v8-e80085-s002.pdf]

Multimedia Appendix 2

Literature Review Search Strategy Results

Primary Search Results

Database: Ovid MEDLINE
ALL <1946 to May 20, 2024>

#	Query	Results from 21 May 2024
1	("Nursing administration" or "Nursing management" or "Leadership in nursing" or "Clinical leadership (in a nursing context)" or "Nursing directors" or "Nursing executives" or "Healthcare leadership (with a focus on nursing)" or "Nurse leadership roles" or "Leadership roles in nursing" or "Nursing supervisors" or "Nursing governance" or "Senior nursing professionals" or "Nurse managers" or "Chief Nursing Officer (CNO) and other executive roles" or "Nursing headship" or "Nursing command" or "Nursing chiefs" or "Nursing officer roles" or "Professional leadership in nursing" or "Charge Nurse" or "Head nurse" or "Nurse leader" or "Nursing leadership").mp. [mp=title, abstract, original title, name of substance word, subject heading word, floating sub-heading word, keyword heading word, organism supplementary concept word, protocol supplementary concept word, rare disease supplementary concept word, unique identifier, synonyms, population supplementary concept word, anatomy supplementary concept word]	14,155
2	limit 1 to (English language and yr.= "2014 - 2025")	5,680
3	("Artificial intelligence" or "AI" or "Machine intelligence" or "Computational intelligence" or "Decision-support systems" or "Automated decision-making" or "Intelligent systems" or "Smart systems" or "AI technology" or "Intelligent automation" or "Robotics and AI" or "Machine learning" or "Deep learning" or "Neural networks" or "Algorithmic intelligence" or "Cognitive computing" or "AI applications" or "AI tools" or "AI solutions" or "Advanced analytics" or "Data science" or "Predictive analytics").mp. [mp=title, abstract, original title, name of substance word, subject heading word, floating sub-heading word, keyword heading word, organism supplementary concept word, protocol supplementary concept word, rare disease supplementary concept word, unique identifier, synonyms, population supplementary concept word, anatomy supplementary concept word]	321,369
4	limit 3 to (English language and yr.= "2014 - 2025")	252,798
5	("Health care" or "Healthcare" or "Medical care" or "Clinical care" or "Patient care" or "Health services" or "Medical services" or "Clinical services" or "Health systems" or "Medical systems" or "Clinical systems" or "Health management" or "Medical management" or "Clinical management" or "Health service delivery" or "Medical service delivery" or "Clinical	1,862,325

	service delivery" or "Healthcare sector" or "Medical industry" or "Health industry").mp. [mp=title, book title, abstract, original title, name of substance word, subject heading word, floating sub-heading word, keyword heading word, organism supplementary concept word, protocol supplementary concept word, rare disease supplementary concept word, unique identifier, synonyms, population supplementary concept word, anatomy supplementary concept word]	
6	limit 5 to (English language and yr.= "2014 - 2025")	835,278
7	2 and 4	52
8	6 and 7	26

("Nursing administration" or "Nursing management" or "Leadership in nursing" or "Clinical leadership (in a nursing context)" or "Nursing directors" or "Nursing executives" or "Healthcare leadership (with a focus on nursing)" or "Nurse leadership roles" or "Leadership roles in nursing" or "Nursing supervisors" or "Nursing governance" or "Senior nursing professionals" or "Nurse managers" or "Chief Nursing Officer (CNO) and other executive roles" or "Nursing headship" or "Nursing command" or "Nursing chiefs" or "Nursing officer roles" or "Professional leadership in nursing" or "Charge Nurse" or "Head nurse" or "Nurse leader" or "Nursing leadership").mp. [mp=title, book title, abstract, original title, name of substance word, subject heading word, floating sub-heading word, keyword heading word, organism supplementary concept word, protocol supplementary concept word, rare disease supplementary concept word, unique identifier, synonyms, population supplementary concept word, anatomy supplementary concept word]
limit 1 to (English language and yr.= "2014 - 2025")
("Artificial intelligence" or "AI" or "Machine intelligence" or "Computational intelligence" or "Decision-support systems" or "Automated decision-making" or "Intelligent systems" or "Smart systems" or "AI technology" or "Intelligent automation" or "Robotics and AI" or "Machine learning" or "Deep learning" or "Neural networks" or "Algorithmic intelligence" or "Cognitive computing" or "AI applications" or "AI tools" or "AI solutions" or "Advanced analytics" or "Data science" or "Predictive analytics").mp. [mp=title, book title, abstract, original title, name of substance word, subject heading word, floating sub-heading word, keyword heading word, organism supplementary concept word, protocol supplementary concept word, rare disease supplementary concept word, unique identifier, synonyms, population supplementary concept word, anatomy supplementary concept word]
limit 3 to (English language and yr.= "2014 - 2025")
("Health care" or "Healthcare" or "Medical care" or "Clinical care" or "Patient care" or "Health services" or "Medical services" or "Clinical services" or "Health systems" or "Medical systems" or "Clinical systems" or "Health management" or "Medical management" or "Clinical management" or "Health service delivery" or "Medical service delivery" or "Clinical service delivery" or "Healthcare sector" or "Medical industry" or "Health industry").mp. [mp=title, book title, abstract, original title, name of substance word, subject heading word, floating sub-heading word, keyword heading word, organism supplementary concept word, protocol supplementary concept word, rare disease supplementary concept word, unique identifier, synonyms, population supplementary concept word, anatomy supplementary concept word]
limit 5 to (English language and yr.= "2014 - 2025")

2 and 4
6 and 7

*Please note that although included in the search string documentation, "book title" were excluded during the screening process in Covidence as per the inclusion criteria.

Secondary Search Results

Database:
Ovid MEDLINE(R) ALL <1946 to May 19, 2025>

#	Query	Results from 19 May 2025
1	("Nursing administration" or "Nursing management" or "Leadership in nursing" or "Clinical leadership (in a nursing context)" or "Nursing directors" or "Nursing executives" or "Healthcare leadership (with a focus on nursing)" or "Nurse leadership roles" or "Leadership roles in nursing" or "Nursing supervisors" or "Nursing governance" or "Senior nursing professionals" or "Nurse managers" or "Chief Nursing Officer (CNO) and other executive roles" or "Nursing headship" or "Nursing command" or "Nursing chiefs" or "Nursing officer roles" or "Professional leadership in nursing" or "Charge Nurse" or "Head nurse" or "Nurse leader" or "Nursing leadership").mp. [mp=title, book title, abstract, original title, name of substance word, subject heading word, floating sub-heading word, keyword heading word, organism supplementary concept word, protocol supplementary concept word, rare disease supplementary concept word, unique identifier, synonyms, population supplementary concept word, anatomy supplementary concept word]	15,088
2	limit 1 to (english language and yr= "2014 -Current")	6,595
3	("Artificial intelligence" or "AI" or "Machine intelligence" or "Computational intelligence" or "Decision-support systems" or "Automated decision-making" or "Intelligent systems" or "Smart systems" or "AI technology" or "Intelligent automation" or "Robotics and AI" or "Machine learning" or "Deep learning" or "Neural networks" or "Algorithmic intelligence" or "Cognitive computing" or "AI applications" or "AI tools" or "AI solutions" or "Advanced analytics" or "Data science" or "Predictive analytics").mp. [mp=title, book title, abstract, original title, name of substance word, subject heading word, floating sub-heading word, keyword heading word, organism supplementary concept word, protocol supplementary concept word, rare disease supplementary concept word, unique identifier, synonyms, population supplementary concept word, anatomy supplementary concept word]	402,858
4	limit 3 to (english language and yr= "2014 -Current")	333,482

5	("Health care" or "Healthcare" or "Medical care" or "Clinical care" or "Patient care" or "Health services" or "Medical services" or "Clinical services" or "Health systems" or "Medical systems" or "Clinical systems" or "Health management" or "Medical management" or "Clinical management" or "Health service delivery" or "Medical service delivery" or "Clinical service delivery" or "Healthcare sector" or "Medical industry" or "Health industry").mp. [mp=title, book title, abstract, original title, name of substance word, subject heading word, floating sub-heading word, keyword heading word, organism supplementary concept word, protocol supplementary concept word, rare disease supplementary concept word, unique identifier, synonyms, population supplementary concept word, anatomy supplementary concept word]	1,987,066
6	limit 5 to (english language and yr= "2014 -Current")	957,191
7	2 and 4	82
8	6 and 7	38

Ovid MEDLINE(R) ALL <1946 to May 16, 2025>

1 ("Nursing administration" or "Nursing management" or "Leadership in nursing" or "Clinical leadership (in a nursing context)" or "Nursing directors" or "Nursing executives" or "Healthcare leadership (with a focus on nursing)" or "Nurse leadership roles" or "Leadership roles in nursing" or "Nursing supervisors" or "Nursing governance" or "Senior nursing professionals" or "Nurse managers" or "Chief Nursing Officer (CNO) and other executive roles" or "Nursing headship" or "Nursing command" or "Nursing chiefs" or "Nursing officer roles" or "Professional leadership in nursing" or "Charge Nurse" or "Head nurse" or "Nurse leader" or "Nursing leadership").mp. [mp=title, book title, abstract, original title, name of substance word, subject heading word, floating sub-heading word, keyword heading word, organism supplementary concept word, protocol supplementary concept word, rare disease supplementary concept word, unique identifier, synonyms, population supplementary concept word, anatomy supplementary concept word] 15088
2 limit 1 to (english language and yr= "2014 -Current") 6595
3 ("Artificial intelligence" or "AI" or "Machine intelligence" or "Computational intelligence" or "Decision-support systems" or "Automated decision-making" or "Intelligent systems" or "Smart systems" or "AI technology" or "Intelligent automation" or "Robotics and AI" or "Machine learning" or "Deep learning" or "Neural networks" or "Algorithmic intelligence" or "Cognitive computing" or "AI applications" or "AI tools" or "AI solutions" or "Advanced analytics" or "Data science" or "Predictive analytics").mp. [mp=title, book title, abstract, original title, name of substance word, subject heading word, floating sub-heading word, keyword heading word, organism supplementary concept word, protocol supplementary concept word, rare disease supplementary concept word, unique identifier, synonyms, population supplementary concept word, anatomy supplementary concept word] 402858
4 limit 3 to (english language and yr= "2014 -Current") 333482
5 ("Health care" or "Healthcare" or "Medical care" or "Clinical care" or "Patient care" or "Health services" or "Medical services" or "Clinical services" or "Health systems" or "Medical

systems" or "Clinical systems" or "Health management" or "Medical management" or "Clinical management" or "Health service delivery" or "Medical service delivery" or "Clinical service delivery" or "Healthcare sector" or "Medical industry" or "Health industry").mp. [mp=title, book title, abstract, original title, name of substance word, subject heading word, floating sub-heading word, keyword heading word, organism supplementary concept word, protocol supplementary concept word, rare disease supplementary concept word, unique identifier, synonyms, population supplementary concept word, anatomy supplementary concept word] 1987066

6 limit 5 to (english language and yr= "2014 -Current") 957191

7 2 and 4 82

8 6 and 7
