# Supplementary material for: Exploring the Intersection of Nursing Leadership and Artificial Intelligence: Scoping Review
Source: JMIR Nurs. 2025 Nov 14;8:e80085. doi: 10.2196/80085 (PMC12617831; doi:10.2196/80085)
Supplement: Multimedia Appendix 3 [file nursing-v8-e80085-s003.pdf]

Multimedia Appendix 3: Overview of Findings

Research Question	Types of Articles (n=26)	Country of Publication	Population	Data Categories
What are the contemporary perspectives on AI technology and nursing leadership?	18 expert commentaries, perspectives, editorials, or theoretical papers [1-11, 20-26]<br><br>**8 empirical studies:** qualitative, quantitative, or mixed methods [12-19]	**Expert commentaries, perspectives, editorials, or theoretical papers (n=18):**<br><br>United States (n=13) [1-11, 20, 22, 24, 26]<br>Canada (n=1) [21]<br>Republic of Korea (n=1) [23]<br>United Kingdom (n=1) [19]<br>Sweden (n=1) [16]<br><br>**Empirical studies (n=8):**<br>Turkey (n=1) [12]<br>Finland (n=1) [13]<br>China (n=1) [14]	**Executive and senior nurse leaders** (chief nursing officers, executives, directors) [1-3, 5-9, 11, 20-23, 24-26]<br><br>**Middle managers and unit-level leaders** (nurse managers, supervisors, charge nurses) [4, 12-15, 17]<br><br>**Clinical nurse specialists** (clinical nurse specialists) [10]<br><br>**Multidisciplinary groups including nurse leaders** (PACU staff, care-home stakeholders, physicians, developers, coordinators) [16, 18, 19]	**Leading digital transformation and technology integration** (n=20)<br>Nurse leaders positioned as central to digital adoption, governance, and workforce planning [1, 2, 8-10, 12-14, 16-22, 24-26].
				**AI technology and the nursing role: reshaping practice** (n=19)<br>AI technology tools described as reducing administrative burden, shifting non-value-added tasks, and enabling proactive leadership [4, 7, 8, 11, 13-17, 19-22, 25, 26].
				**Ethical considerations of AI technology for nurse leaders** (n=12)<br>Concerns regarding autonomy, bias, safety, and governance highlight the need for leaders to safeguard equity and ethical practice [1, 2, 6, 10, 11, 17, 19-22, 24, 26].
				**AI technology as a facilitator of innovative leadership** (n=15)<br>AI technology identified as a catalyst for innovation, leadership self-efficacy, and transformative practices [3, 5, 7-9, 11, 12, 14, 17, 20-22, 24, 26].
				**Education and training on AI technology in nursing practice** (n=15)<br>Articles emphasized digital literacy, continuous education, and leadership preparation to ensure readiness [1, 2, 4, 5, 7-11, 13, 14, 17, 21, 24-26].

		Australia (n=1) [15]<br>Sweden (n=2) [16-18]<br>Egypt (n=1) [17]<br>United Kingdom (n=1) [19]		**Influence of AI technology on the work environment** (n=12)<br>Predicted effects include reduced burnout, efficiency gains, and reshaping of relational care in acute and long-term settings [1, 3, 4, 8, 9, 11, 14, 16, 17, 19, 24, 25].

References:

1. Blouin, AS. Innovations in nursing workforce management: integrating emerging technologies with proven strategies. *The Journal of Nursing Administration* 2023;53(11):601-606 doi:10.1097/NNA.0000000000001352
2. Cineas, N, Schwartz, DB, Patel, K. Moving forward into the future: nurses and nurse leaders at leading us public health system implementing foundational transformation to advance health equity. *Nursing Administration Quarterly* 2022;46(3):E44-E50 doi:10.1097/NAQ.0000000000000539
3. Clancy, TR. Technology solutions for nurse leaders. *Nursing Administration Quarterly* 2020;44(4):300-315 doi:10.1097/NAQ.0000000000000439
4. Clipper, B, Batcheller, J, Thomaz, AL, Rozga, A. Artificial intelligence and robotics: a nurse leader's primer. *Nurse Leader* 2018;16(6):379-384 doi:10.1016/j.mnl.2018.07.015
5. Douglas, K, Gray, S. Generational complexities present new challenges for nurse leaders. *Nurse Leader* 2020;18(2):126-129 doi:10.1016/j.mnl.2019.12.008
6. Fontenot, J. Spotlight on leadership: what nurse leaders need to know about artificial intelligence. *The Journal of Nursing Administration* 2024;54(2):74-76 doi:10.1097/NNA.0000000000001384
7. Fuller, R, Hansen, A. Navigating and leading the future of nursing. *Nursing Administration Quarterly* 2019;43(3):212-221 doi:10.1097/NAQ.0000000000000354

8. Porter-O'Grady, T. Turning the page: nursing in the digital age and beyond. *Nursing Management* 2019;50(9):40-47 doi:10.1097/01.NUMA.0000579012.32858.2b
9. Siedlecki, SL. Artificial intelligence, digital health research, and the clinical nurse specialist. *Clinical Nurse Specialist: The Journal for Advanced Nursing Practice* 2023;37(5):214-217 doi:10.1097/NUR.0000000000000763
10. Sullivan, D, Hall, VP, Morrison, J. Navigating the future: artificial intelligence's impact on transformational nurse leadership. *Teaching and Learning in Nursing* 2024;19(3):298-300 doi:10.1016/j.teln.2024.04.017
11. Eminoğlu, A, Çelikkanat, Ş. Assessment of the relationship between executive nurses' leadership self-efficacy and medical artificial intelligence readiness. *International Journal of Medical Informatics* 2024;184:105386 doi:10.1016/j.ijmedinf.2024.105386
12. Laukka, E, Hammarén, M, Kanste, O. Nurse leaders' and digital service developers' perceptions of the future role of artificial intelligence in specialized medical care: an interview study. *Journal of Nursing Management* 2022;30(8):3838-3846 doi:10.1111/jonm.13769
13. Li, X, Cheng, M, Xu, J. Leaders' innovation expectation and nurses' innovation behaviour in conjunction with artificial intelligence: the chain mediation of job control and creative self-efficacy. *Journal of Nursing Management* 2022;30(8):3806-3816 doi:10.1111/jonm.13749
14. El-Gazar, HE, Shawer, M, Alenezi, A, Shaban, M, Zaky, ME, Zoromba, MA. Strengths mindset as a mediator in the relationship between paradoxical leadership and nurses' positive attitudes towards artificial intelligence: a cross-sectional study. *Journal of Advanced Nursing* 2025;(7609811, h3l) doi:10.1111/jan.16841
15. Finkelstein, J, Gabriel, A, Schmer, S, Truong, TT, Dunn, A. Identifying facilitators and barriers to implementation of ai-assisted clinical decision support in an electronic health record system. *Journal of Medical Systems* 2024;48(1):1-23 doi:10.1007/s10916-024-02104-9
16. Kotp, MH, Ismail, HA, Basyouny, HAA, et al. Empowering nurse leaders: readiness for ai integration and the perceived benefits of predictive analytics. *BMC Nursing* 2025;24(1):56 doi:10.1186/s12912-024-02653-x
17. Lundsten, S, Jacobsson, M, Ryden, P, Mattsson, L, Lindgren, L. Using ai to predict patients' length of stay: pacu staff's needs and expectations for developing and implementing an ai system. *Journal of Nursing Management* 2024;2024(bzy, 9306050):3189531 doi:10.1155/jonm/3189531
18. Neves, BB, Omori, M, Petersen, A, Vered, M, Carter, A. Navigating artificial intelligence in care homes: competing stakeholder views of trust and logics of care. *Social Science & Medicine* 2024;358:N.PAG-N.PAG doi:10.1016/j.socscimed.2024.117187

19. Aldrich, K, Chipps, E, Mook, PJ. Driving innovations: nursing leadership think tank explores ai solutions. *Nurse Leader* 2025 doi:10.1016/j.mnl.2025.04.001
20. Cardiff, B, Irani, CSS, Risling, T, et al. From federal policy to bedside care: a canadian perspective on key considerations for nurse executive action for ai adoption - a commentary. *Nursing Leadership* 2025;37(3):48-66 doi:10.12927/cjnl.2025.27509
21. Hoelscher, SH, Taylor-Pearson, K, Wei, H. Charting the path: nursing leadership in artificial intelligence integration into healthcare. *Nurse Leader* 2024;22(6):763-772 doi:10.1016/j.mnl.2024.07.011
22. Park, CSY. Ethical artificial intelligence in nursing workforce management and policymaking: bridging philosophy and practice. *Journal of Nursing Management* 2025;2025(bzy, 9306050):7954013 doi:10.1155/jonm/7954013
23. Sloss, E, Austin, R, Kennedy, R, Carter-Templeton, H. ANI emerging leader project: engaging nurses to drive the implementation of artificial intelligence in nursing practice. *Computers, Informatics, Nursing* 2025;43(3):1-1 doi:10.1097/CIN.0000000000001201
24. Tyransky, CE, Paulus, K, Langmead, E, et al. Integration of virtual technology and artificial intelligence improves satisfaction, patient safety, and nursing workforce efficiency. *Journal of Nursing Care Quality* 2025 doi:10.1097/NCQ.0000000000000842
25. Virkstis, K. The role of artificial intelligence in supporting the core mission of nursing. *The Journal of Nursing Administration* 2025;55(3):135-137 doi:10.1097/NNA.0000000000001544
26. Stucky, C, Wymer, J. Ethical reasoning as a core nurse leader competency: maximizing strategic decision-making and operational outcomes. *Nurse Leader* 2024:187-193 doi:10.1016/j.mnl.2023.09.009
